# Editorial: Crosstalk between epigenetics on the development of cancer and cardiovascular disease

**DOI:** 10.3389/fcell.2022.1027798

**Published:** 2022-10-20

**Authors:** Donghong Zhang, Yidong Wang, Tharmarajan Ramprasath, Ping Wang, Rodolfo Negri

**Affiliations:** ^1^ Department of Pharmacology and Chemical Biology, Winship Cancer Institute, Emory University School of Medicine, Atlanta, GA, United States; ^2^ School of Basic Medical Sciences, Institute of Cardiovascular Sciences, Xi’an Jiaotong University Health Science Center, Xi’an, China; ^3^ Center for Molecular and Translational Medicine, Georgia State University, Atlanta, GA, United States; ^4^ School of Medical Imaging, Tianjin Medical University, Tianjin, China; ^5^ Department of Biology and Biotechnology “C.Darwin”, Sapienza University of Rome, Rome, Italy

**Keywords:** epigenetics, cancer, cardiovascular disease, epigenetic modification, epigenetic drug

## Introduction

Chromatin structure is fundamental for transcription regulation (McGinty and Tan, 2015) and genome integrity (Groth et al., 2007; Di Nisio et al., 2021). It can be modified by very complex and variegated machinery with major effects on cell biology. In the last 20 years, several basic epigenetic mechanisms have been described to critically regulate genome expression and preservation while significant advances have been made in identifying and characterizing the complex biochemical machinery involved. These efforts are mainly due to the understanding of the prominent roles of epigenetic modifications in physiological and pathological states. Indeed, an impressive body of experimental evidence has shown the involvement of mutations or dysregulation of epigenetic modifiers in human diseases. While this concept was clear from the beginning in cancer research (Morgan and Shilatifard 2015), it is now rapidly extending to non-cancer diseases such as neurodegenerative syndromes and cardiovascular dysfunctions.

## Crosstalk between epigenetics on the development of cancer

All the main epigenetic mechanisms (DNA methylation; histone modification; nucleosome remodeling; mRNA modification and noncoding RNA-mediated processes) seem to be involved in cancer development. Dysfunctions in epigenetic machinery may easily cause genetic instability, which can be inherited from mother to daughter cells, steering cell lineages toward increasingly pathological states. There is consequently great interest in epigenetic drugs, which are potentially capable of counteracting the deleterious effects of the dysfunctions by blocking or supporting the affected modifications. Although very promising, the pharmacological progress in this field is facing great difficulties (Wimalasena et al., 2020; Asano, 2020):

i) epigenetic modifications have typically pleiotropic effects and interfering with them can cause major out-target effects; and
ii) the complexity of the epigenetic machinery makes it very difficult to specifically inhibit single epigenetic actors without affecting structurally similar proteins with distinct or sometimes even opposite functions.

## The role of epigenetics in specific cancer diseases

Several excellent reviews have been published on the general role of epigenetic mechanisms in cancer (Dawson and Kouzarides, 2012) while, more recently, the scientific community is focusing on specific cancer diseases with the major goal to identify potential diagnostic biomarkers and/or putative experimental therapy approaches (Xue et al., 2022; Zhang et al., 2022). Testicular cancer is an excellent example of this line of research. Most testicular cancers are testicular germ cell tumors (TGCTs), which can be classified into seminomas (SGCTs) and non-seminoma testicular germ cell tumors (NSGCTs). During their development, primordial germ cells (PGCs) undergo epigenetic modifications and any dysfunctions in their pattern might lead to cancer development. In this special issue, Nicu et al. provide a comprehensive review of the epigenetic mechanisms potentially involved. They include DNA methylation, histone modifications, and non-coding RNAs associated with TGCT susceptibility, initiation, progression, and response to chemotherapy. The authors also review the progresses in the identification and development of epigenetic biomarkers as powerful tools for the diagnostics and prognostics, and the efforts to develop epigenetic-based therapies.

## Emerging epigenetic mechanisms

New emerging epigenetic mechanisms involved in cancer development should also be further explored and characterized. Among them, the most interesting one is certainly mRNA N6-adenosine methylation (m6A). One of the most important genes involved in this modification is METTL3 (Wei et al., 2022) which encodes the 70 kDa subunit of MT-A, which is part of N6-adenosine-methyltransferase. This enzyme is involved in the posttranscriptional methylation of internal adenosine residues in eukaryotic mRNAs, forming N6-methyladenosine. A very thought-provoking study by Jiang et al. in this special issue provides a comprehensive Pan-Cancer analysis of the prognostic and immunological roles of the METTL3/lncRNA-SNHG1/miRNA-140-3p/UBE2C axis and suggests that this axis can be a prognostic indicator, as well as a promising therapeutic target, for patients with non-small cell lung cancer (NSCLC). In the same issue, Fang et al*.* present a transcriptome analysis of RNA N6-Methyladenosine modification in adriamycin-resistant Acute Myeloid Leukemia (AML) Cells and suggest that quantitative and qualitative modulation of this modification plays an important role in adriamycin-resistant AML.

## The role of epigenetics in the development of cardiovascular disease

In contrast to the intensive research into the roles of epigenetics in cancers, much less progress has been made in non-cancer diseases, while cardiovascular disease has recently become one of the most advanced frontiers. The most intensive crosstalk between various epigenetic modifications has been found in multiple cardiovascular disease. For instance, m6A modification collaborates with specific microRNAs or piRNAs to regulate cardiac development and hypertrophy (Gao et al., 2020; Qian et al., 2021). N6-methyladenine (m6A) DNA-regulated lncRNA myocardial infarction-associated transcript (MIAT) promotes plaque progression in atherosclerosis (Wu et al., 2019). In this special issue, Chen et al., report a comprehensive review on the latest progress on the epigenetic regulation of myocardial infarction, focusing on DNA methylation, histone modifications, and microRNA-dependent mechanisms. The authors also discuss the novel therapies based on epigenetics.
